# Tenofovir use is associated with low vitamin D levels in a Spanish HIV cohort

**DOI:** 10.1186/1758-2652-13-S4-P104

**Published:** 2010-11-08

**Authors:** M Cervero, C García Lacalle, JL Agud, R Torres Perea, E García-Benaya, J Jusdado, C Pérez Pons, M del Álamo

**Affiliations:** 1 H. Severo Ochoa, Internal Medicine. Infectious Disease Unit, Madrid, Spain; 2 H. Severo Ochoa, Biochemist Service, Madrid, Spain; 3 H. Severo Ochoa, Internal Medicine, Madrid, Spain; 4 H. Severo Ochoa, Pharmacy Service, Madrid, Spain; 5 H. Severo Ochoa, Hematology Service, Madrid, Spain; 6 H. Severo Ochoa, Microbiology Service, Madrid, Spain

## Background

Several studies have shown a high frequency of vitamin D deficiency among HIV patients. Several studies have ascribed these low levels of vitamin D to antiretroviral treatment, especially efavirenz. Tenofovir has been related to changes of bone mineralization in SIV-infected rhesus monkeys and with loss of bone mineral density in children. Adequate vitamin D stores have the potential of an improvement of immune status, lessening of cardiovascular risk and beneficial effects on certain neoplastic disorders.

## Methods

Cross-sectional study of 94 adult HIV outpatients in Leganés (Madrid, Spain) performed in 2008. Risk factors for vitamin D deficiency (< 20 µg/L) were examined using logistic regression.

## Results

Median age was 44 years (IQR 40 to 48); 69.1% were males, 93,6% whites, 6.4% black race. Mean CD4+ cell count was 446 cells/µL (IQR 312 to 586). Viral load was below 50 copies/mL in 78.7%. Median 25(OH)D level was 17.7 µg/L (IQR 11.9 TO 24.3). 87.2% of patients had 25(OH)D < 30 µg/L (suboptimal), 57.4% had 25(OH)D < 20 µg/L (deficient ) and 19.1% < 10 µg/L (severely deficient). Factors associated with low levels of 25(OH)D were heterosexual vs. IVDU HIV-risk group (OR 13.3, 95% CI 2.4-74.1, p=0.003), season (spring vs. summer; OR 16.8, 95% CI 3.4-82.1, p=0.0001), age >45 vs < 45 years (OR 10.5, 95% CI 2.4-46.6%, p=0.002), CD4+ cells nadir <200 vs >200 cells/µL (OR 4.1, 95% CI 1.01-17.6, p=0.049), and tenofovir vs. abacavir therapy (OR 12.7, 95% CI 1.8-87.1, p=0.01). Black race is underrepresented to draw conclusions. In this sample, no association of low 25(OH)D with efavirenz was found.

## Conclusions

Despite low latitude, low levels of vitamin D are almost universal in our sample of HIV outpatients with satisfactory immunologic and virologic response to ART. Increasing age, less insolation season, heterosexual risk group, and CD4+ nadir were associated with lower levels. Tenofovir use was associated with lower levels of 25(OH)D. Further studies on causality of this association and the need of control bone-mineral density in tenofovir-treated patients seems warranted.

